# A case of giant sarcomatoid carcinoma ex-pleomorphic adenoma of the parotid gland

**DOI:** 10.11604/pamj.2020.37.2.21653

**Published:** 2020-09-01

**Authors:** Mehdi Hasnaoui, Mohamed Masmoudi, Nouha Ben Abdeljelil, Takwa Belaid, Khalifa Mighri

**Affiliations:** 1 Department of Otolaryngology-Head and Neck Surgery, Tahar Sfar Hospital, Mahdia, Tunisia,; 2 Department of Pathology, Fattouma Bourguiba Hospital, Monastir, Tunisia

**Keywords:** Parotid neoplasms, carcinoma, pleomorphic adenoma

## Abstract

Carcinoma ex pleomorphic adenoma is a rare malignant parotid neoplasm arising from primary or recurrent benign pleomorphic adenoma. Histologically, it can be sub-classified by upwards of eight different variations (e.g., myoepithelial carcinoma, salivary duct carcinoma, adenoid cystic carcinoma sarcomatoid carcinoma). We present the fourth case in the literature of sarcomatoid carcinoma arising from an underlying pleomorphic adenoma of the parotid gland. We present a case of a 47-year-old female who consulted for a left parotid mass of 15cm long axis. There was no facial paralysis. Fine needle aspiration cytology smears showed a pleomorphic adenoma. The parotid MRI showed a left parotid mass, with heterogeneous signal (hyposignal T1 and hypersignal T2 and in diffusion sequences). The patient underwent a total parotidectomy with a pleomorphic adenoma on extemporaneous examination. Histological examination of the part revealed a pleomorphic adenoma on which a sarcomatoid carcinoma developed. Therefore, a second operation occurred. We performed selective lymph node dissection carrying out the sectors I, II and III followed by radiotherapy. The evolution was favorable. In addition to its rarity, our case joins historical cases by its huge size.

## Introduction

Carcinoma ex pleomorphic adenoma (Ca ex PA) is an epithelial malignancy that arises from a pre-existing pleomorphic adenoma (PA) [1]. Ca ex PA generally arises from the parotid gland (29-82% of all cases) [2]. Among all salivary gland neoplasms, Ca ex PA accounts for approximately 3.6% [3,4]. Histologically, it can be sub-classified by upwards of eight different variations (e.g., myoepithelial carcinoma, salivary duct carcinoma, adenoid cystic carcinoma sarcomatoid carcinoma) [5]. We present the fourth case in the literature of sarcomatoid carcinoma arising from an underlying pleomorphic adenoma of the parotid gland.

## Patient and observation

**Figure 1:**
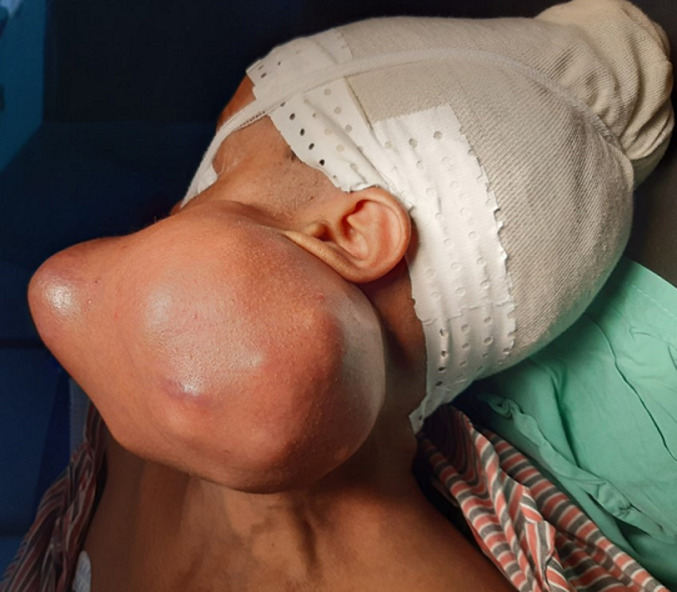
side photo showing a left parotid mass of 15 cm long axis with a bumpy surface and peeling off the ear lobule. The skin next to the swelling was healthy

**Figure 2:**
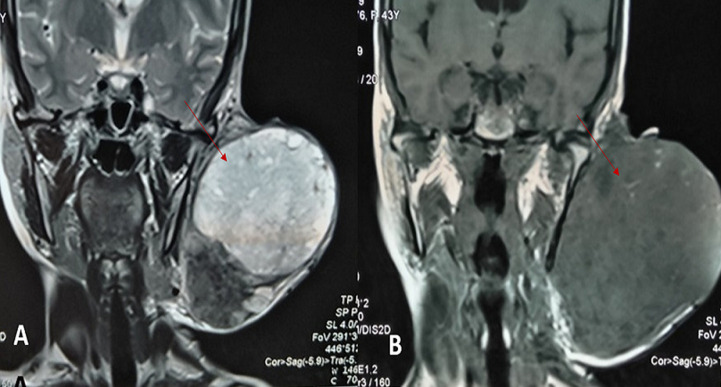
parotid MRI in axial sections showing a left parotid mass in T2 hypersignal A) and T1 hyposignal B)

**Figure 3:**
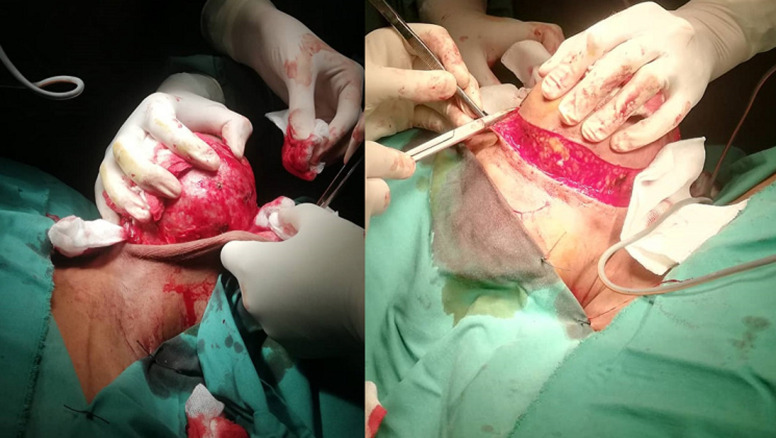
intraoperative view showing subcutaneous separation and tumor exposure

**Figure 4:**
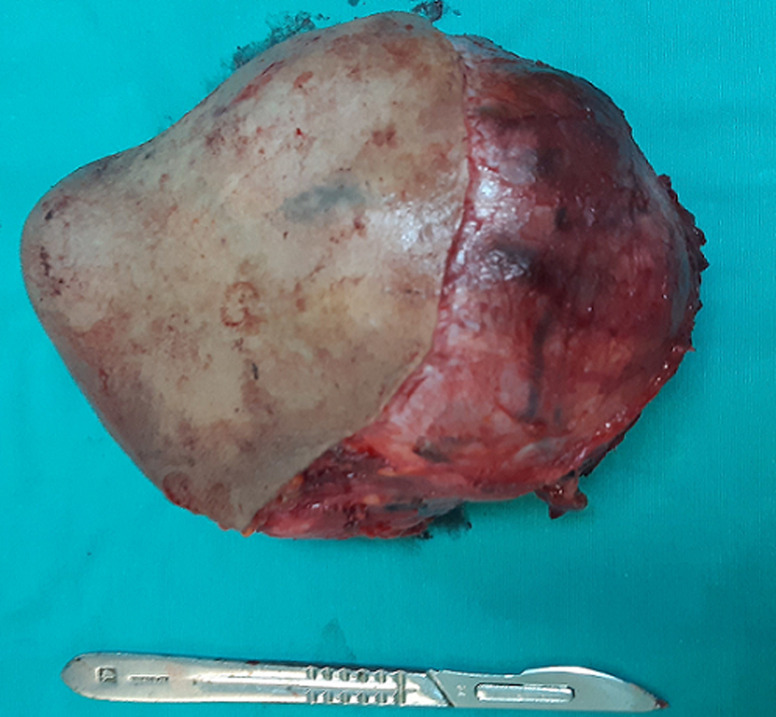
aspect of excised tumoural formation. It measured 15 cm in large diameter

**Figure 5:**
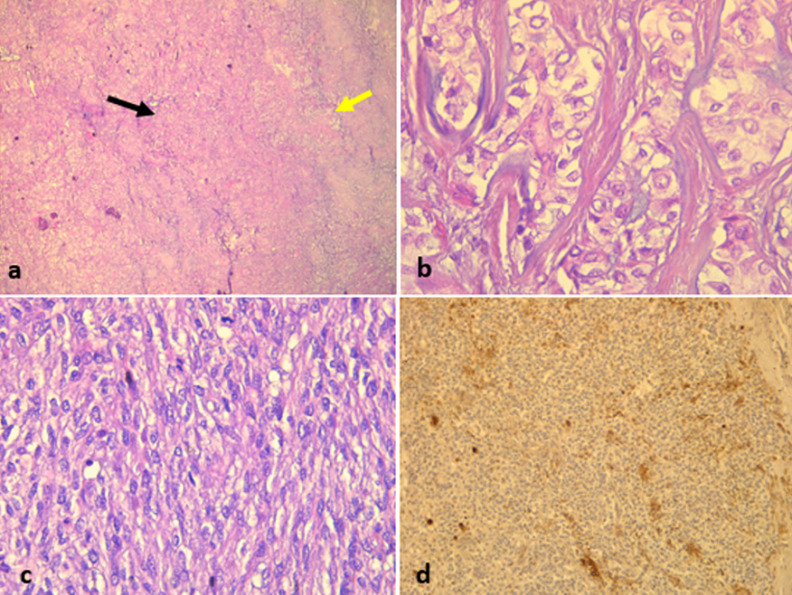
A) abrupt transition from pleomorphic adenoma (yellow arrow at right) to sarcomatoid carcinoma (black arrow at left), HEx40; B) epithelial myoepithelial carcinoma formed by ducts or tubules with an outer rim of myoepithelial cells and inner, dark ductal cells with scant eosinophilic cytoplasm and round, bland nuclei (HEx400); C) spindle cells showing mild to marked nuclear atypia with frequent mitotic figures HEx 400; D) spindle cells are focally positive with AE1/AE3

**Figure 6:**
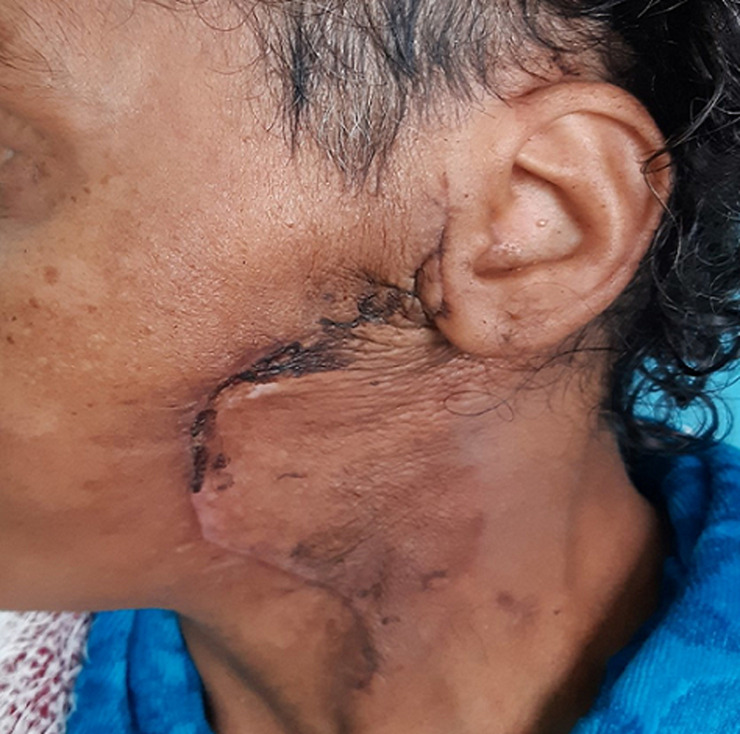
patient 7 months after the surgery. There were no swellings in the left parotid region

She was a 47 year old woman. She had no particular pathological history. She consulted for a parotid swelling evolving for 2 years, gradually increasing in size. A rapid increase in size of the swelling has been noted for 3 months. Physical examination found a left parotid mass of 15cm long axis, firm, with a bumpy surface and peeling off the ear lobule. However, the skin next to the swelling was healthy (Figure 1). There was no facial asymmetry, no lockjaw and no palpable cervical lymphadenopathies. The ostium of the Stenon duct were free with an issue of clear saliva. The parotid MRI objectified a left parotid mass, with heterogeneous signal (hyposignal T1 and hypersignal T2 and in diffusion sequences).This mass measured 15cm in large diameter and it was located in the superficial lobe of the parotid. It remained distant from the vascular axis. No cervical lymphadenopathy was identified in this exam (Figure 2). A FNAC was carried out concluding with a pleomorphic adenoma. The patient had a total parotidectomy with a pleomorphic adenoma on extemporaneous examination. Intraoperatively, the skin was intact. The tumor was located in the superficial lobe of the parotid. A simple skin suture was performed without using a flap (Figure 3, Figure 4). Histological examination revealed a sarcomatoid carcinoma ex pleomorphic adenoma. Indeed the malignant componant was made of tubes and nests of tumors cells. The tubes were lined with an outer rim of myoepithelial cells and inner, dark ductal cells with scant eosinophilic cytoplasm and round, bland nuclei. On some areas, the tumor was formed by spindle cells which showed mild to marked nuclear atypia with frequent mitosis. Immunohistochemically, epithelial cells were positive with AE1/AE3, cytokeratin7 and epithelial membrane antigen (EMA). Myoepithelial cells expressed myoepithelial markers (PS100 and P63). Spindle cells were negative with PS100, smooth muscle actin, p63 and were focally positive with AE1/AE3 (Figure 5). The patient had a surgery for the second time. We performed selective lymph node dissection carrying out the sectors I, II and III. The lymphadenopathies were non-metastatic and the radiological examinations didn´t reveal any metastases. Then, the patient had a radiotherapy. The patient evolved well after treatment. We didn´t notice any recurrence after 7 months of surveillance (Figure 6).

## Discussion

Carcinomatous transformation of a pleomorphic adenoma may progress into three different ways: carcinosarcomas, carcinoma ex pleomorphic adenoma, and metastasizing pleomorphic adenomas [6]. The primary distinction behind the first two ultimately lies on the extent of malignancy within its tissue components. Whereas both stromal and epithelial cells have malignant properties in carcinosarcomas, this is often just limited to epithelial components in CXPA. The sarcomatous component is usually the dominant feature in carcinosarcomas and typically is represented by chondrosarcoma [6]. There is a paucity of literature describing the molecular differences between types of CXPA. In one major review of 73 cases of CXPA, Lewis *et al*. found the most common classifications were adenocarcinoma not otherwise specified (NOS, 31 cases, 44%) and salivary duct carcinoma (24 cases, 34%). Least common were epithelial-myoepithelial carcinoma (1 case, 1%) and sarcomatoid carcinoma (1 case, 1%) [5]. Similar numbers were reported in another review of 36 cases by Mariano *et al*. with higher number of salivary duct carcinoma (15 cases, 42%) and just one case of sarcomatoid carcinoma (2.8%) [7]. Another case of sarcomatoid carcinoma has been reported by Parra -Ferro *et al*. [8]. There have reports of sarcomatoid carcinomas (i.e. spindle cell tumors with positive EMA and vimentin markers) arising in different organs throughout the body [9]. However, this is the fourth case of a sarcomatoid carcinoma arising from an underlying pleomorphic adenoma of the parotid gland which has been reported. Generally, Ca ex PA is a neoplasm that affects older patients (mean age, 58 years), with a wide age range of 14-87 years. There is no sex difference [10]. Ca ex PA can be asymptomatic as most of the cancers that are not widely invasive and they often have similar clinical presentations as PA. Symptoms are usually rare or insignificant, most commonly presenting as asymptomatic gradual swelling without facial nerve involvement, a nonspecific finding seen in most parotid masses.

Rapid growth, change in consistency, pain and onset of facial nerve deficit are signs of carcinomatous transformation, the incidence of which increases with duration of a known PA [10,11]. In our case, the tumor had a lateral development outside the facial nerve which explains its asymptomatic character despite its large size. The only sign that drew attention to the malignancy was the rapid increase in size. Ca ex PA may reach, after a long evolution, significant dimensions. According to an international classification of the most voluminous parotid tumors, the largest tumor was operated on by Frylinck in 1956, weighting 26.5kg [12]. Our case joins this historic case, by its huge size. In the case reported by Parra-Ferro, the size of the tumor was 3cm. We do not have detailed information on the other two cases reported in the literature. Several previous series have failed to identify an association between overall tumor size and patient outcome. In our patient, the evolution was good despite the large size of the tumor which measured 15 cm long. The prognosis of Ca ex PA depends on pathological staging parameters like the level of invasion, lymph node involvement, and distant metastasis. Furthermore, grade and the completeness of the tumour resection are also noted to be significant prognostic indicator in invasive Ca ex PA. The histological subtype of Ca ex PA has not been reported as a prognostic factor. The treatment of Ca ex PA often involves an ablative surgical procedure which either may or may not be followed by reconstructive surgery [11]. In the case of Parra-Ferro, a radical parotidectomy with cable nerve graft reconstruction of the buccal branch of facial nerve, neck dissection (levels I-V), and wide local excision of cheek skin was performed. The defect was closed with advancement flaps. In our case we did not use a flap despite the large size of the tumor. In all parotid cancers, the indication for radiotherapy depends on the stage and grade of the tumor and the quality of the surgical excision. Our patient had radiation therapy as she was in stage III.

## Conclusion

This is the fourth case of a sarcomatoid carcinoma arising from an underlying pleomorphic adenoma of the parotid gland which has been reported. We cannot draw any conclusions given the low number of cases reported in the literature. In addition to its rarity, our case joins historical cases by its huge size.
